# A Study on the Effect of Neurogenesis and Regulation of GSK3*β*/PP2A Expression in Acupuncture Treatment of Neural Functional Damage Caused by Focal Ischemia in MCAO Rats

**DOI:** 10.1155/2014/962343

**Published:** 2014-07-10

**Authors:** Ding Luo, Xiaonong Fan, Congcong Ma, Tongtao Fan, Xiaoguang Wang, Nvzi Chang, Lingxin Li, Yanan Zhang, Zhihong Meng, Shu Wang, Xuemin Shi

**Affiliations:** ^1^Postgraduate Department of Tianjin University of Traditional Chinese Medicine, Tianjin 300193, China; ^2^The Acupuncture & Moxibustion Institute, First Teaching Hospital of Tianjin University of Traditional Chinese Medicine, Tianjin 300193, China; ^3^The Third-Level Acupuncture Dose-Effect Laboratory of the State Administration of Traditional Chinese Medicine, Tianjin 300193, China; ^4^Tianjin Key Laboratory of Acupuncture & Moxibustion Science, Tianjin 300193, China; ^5^Department of Rehabilitation Medicine, West China Hospital, Sichuan University, Chengdu, Sichuan 610041, China; ^6^Department of Acupuncture and Moxibustion, First Teaching Hospital of Tianjin University of Traditional Chinese Medicine, Tianjin 300193, China

## Abstract

170 SD rats were randomly divided to five groups. Rats in model group, no-acupuncture group, and acupuncture group were subjected to MCAO surgery. Acupuncture group received 3 consecutive acupuncture treatments at a parameter that deep in 2 mm towards apex nasi and thrust/lifted at 3 times per second for 1 minute, while model group and no-acupuncture group were no-intervention control groups. Serious neural functional damage and sharp decrease of cerebral blood flow, obvious infarction volume, increased nestin mRNA expression, and immunopositive cells population (nestin^+^, BrdU^+^ and nestin/BrdU^+^) were found in MCAO rats which had not been observed in normal group and sham-operated group. However, the damage was attenuated by rat's “self-healing” capacity 3 days after MCAO. And the “self-healing” capacity can be strengthen by acupuncture treatment through increasing cerebral blood flow, neurogenesis, and regulation of gene transcription or GSK-3*β* and PP2A expression. In conclusion, the present study indicates that the underlying mechanism of acupuncture treatment on neural functional damage caused by focal ischemia injury is a multiple interaction which may involve improved cerebral blood supply, neurogenesis, and regulation of gene transcription or GSK-3*β* and PP2A expression in MCAO rats.

## 1. Introduction

Stroke is the leading cause of death and disability and thus a tough challenge for healthcare system and a heavy social burden in China [1]. It is characteristic of ischemia accounting for 43% to 79% of stroke and a belt of high incidence existed in many provinces [2]. Limited by the short time window for thrombolytic therapy, high hopes have been placed on neuron plasticity for treating stroke caused by ischemia. As evidence supplied by animal researches, endogenous neurogenesis occurs in subventricular zone (SVZ) [3] and dentate gyrus of subgranular zone (SGZ) in hippocampus [4–6] throughout mammalian life, which can be triggered by many factors such as ischemic insult [7], traumatic injury [8], apoptosis [9], VEGA [10], and inflammation [11]. These neural stem cells are capable of self-renewal and differentiation that may compensate the damaged neurons and neurogliocytes for subsequent functional recovery, which can be marked by special cellular marker protein such as nestin. Thus, treatment which can amplify endogenous neurogenesis should have great potential for ischemic stroke treatment.

Acupuncture is a traditional therapy derived from ancient China, well-known by its good therapeutic effects on many diseases with long period of large body clinic practice. According to a randomized controlled prospective clinic trial conducted by our team [12], acupuncture effectively improves the self-care ability and life quality of patients with onset of ischemic stroke. Coincidences with the result of clinic trial, animal researches also demonstrate a neuroprotective effect on MCAO rats treated by acupuncture, which is relative to increased brain blood flow, reduced ischemic infarction volume, and lowered neuronal cell death rate in acupuncture group under the proper stimulated parameter on special acupoint [13, 14].

However, the mechanism underlying about how acupuncture works is still unknown. We, therefore, try to reveal the mechanism from accessing neural function deficit, infarction volume, brain blood flow, neural stem cell population, and marker gene expression by neural deficit score, 2,3,5-triphenyl tetrazolium chloride staining, laser-Doppler flow meter, immunofluorenscence double-staining, and real-time polymerase chain reaction. In addition, we also utilize microarray to identify gene profile of acupuncture treatment and enzyme-linked immunoabsorbent assay (ELISA) to verify microarray results. The study aims at elucidating the exact mechanism of acupuncture treatment on ischemia stroke.

## 2. Materials and Methods

### 2.1. Animals and Groups

**Figure 1:**
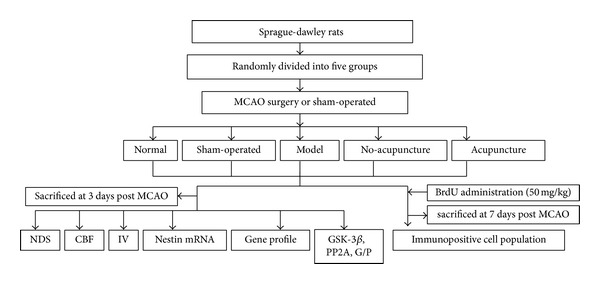
Schematic diagram for methodologies: a total of 170 adult male SD rats were randomly divided into normal, sham-operated, model, no-acupuncture, and acupuncture group. Rats in model group euthanatized immediately after MCAO while other groups at 3 days for subsequent investigation of neural deficit score (NDS), cerebral blood flow (CBF), nestin mRNA expression, microarray analysis, and enzyme-linked immunoabsorbent assay. Another sets of rats sacrificed 7 days for immunopositive cell counting.

A total of 170 adult male Sprague-Dawley rats which were purchased from Experimental Animal Center of Academy of Military Medical Sciences weighting from 250 to 280 g were employed. All animals were housed in a conditioned environment (12 hour-light/12 hour-dark circle, humidity 55 ± 5%, and free access to food and water). All the experimental protocols were approved by local animal ethical committee and consistent with experimental animal use guidelines. Rats were randomly divided into five groups as normal group, sham-operated group, model group, no-acupuncture group, and acupuncture group. Rats in model group, no-acupuncture group, and acupuncture group subjected to MCAO surgery. Acupuncture group received 3 consecutive acupuncture treatments. The model group and no-acupuncture group were no-intervention control groups for acupuncture group. Rats in model group decapitated immediately after behavior measurement by neural deficit scores when recovering consciousness from MCAO surgery. But rats in no-acupuncture group were killed at the same time point as acupuncture group and the difference between two groups was received acupuncture treatment or not (one set sacrificed at 3 days after MCAO for CBF, IV, PCR, and gene microarray measurement while the other set killed at 7 days for immunopositive population counting). It was meant to investigate the immediate damage caused by ischemia and the “self-repairing” ability of MCAO rats. The experimental protocols were shown in Figure 1.

### 2.2. Permanent Middle Cerebral Artery Occlusion

Permanent middle cerebral artery occlusion was performed as previously described [13, 15]. In brief, rats were anesthetized with 10% chloral hydrate (250 mg/kg) by intraperitoneal injection. A 2 to 2.5 cm incision was cut in the centre of neck. Left common carotid artery (CCA), external carotid artery (ECA), and internal carotid artery (ICA) were exposed and isolated. After CCA was clamped and ECA was ligatured by 0^#^ suture, a nylon filament (diameter 0.265 mm) with round tip made by heating near a flame was inserted intraluminally into the ICA about 18 to 20 mm until a slight resistance was felt (which means the tip of the filament reached the origin of middle cerebral artery) and ligatured. Then, we released the clamp in CCA and sutured the incision. Gentamicin was used for anti-infection in incision after surgery. Sham-operated group received the same surgery but without nylon filament insertion. The rats' rectum temperatures were maintained at 37 ± 1°C during the surgery by an electric blanket.

### 2.3. 5′-Bromo-2′-deoxyuridine (BrdU) Administration and Tissue Preparation

BrdU, a thymidine analog that can be integrated into DNA synthesis during S-phase of the cell circle, was employed for capturing the proliferated cell population after ischemic infarction. BrdU (Sigma, USA) was dissolved in 40–50°C warm saline (0.9% NaCl in sterile H_2_O). Rats from each group received fresh dissolved BrdU solution injection intraperitoneally at a dose of 50 mg/kg [16] for 7 consecutive days twice daily 2 hours after the onset of the permanent middle cerebral artery occlusion. But rats in model group only received one BrdU injection before euthanasia. Then rats were euthanasized under over anesthesia, followed by transcardially perfusing with saline and 4% paraformaldehyde in 0.1 M phosphate-buffer at 4°C. Then brains were quickly removed and post-fixed in 12% formalin solution for 24 hours at room temperature. A 3 mm coronal block was cut at 3 mm posterior of optic chiasma and embedded in paraffin, then sectioned at 4 *μ*m each. Sections were dried off at 60°C after immersing in polylysine and prepared for subsequent examination of immunofluorescence double-staining.

### 2.4. Acupuncture Stimulation

Rats in acupuncture group received acupuncture stimulation in consciousness once daily for 3 consecutive days immediately after MCAO performed by a skilled acupuncture practitioner. The DU26 (Shuigou acupoint) located at the junction of the upper one-third and lower two-thirds of the cleft lip midline beneath the nasal septum as described in our previous paper [13] was stimulated by a stainless needle of 0.25 mm in diameter and 30 mm in length (Hua Tuo Medical Instruments Co. Ltd Suzhou, China). The needle was inserted deeply in 2 mm manually towards apex nasi and thrust/lifted stimulated at a parameter of 3 times per second for 1 minute.

### 2.5. Behavioral Measurements

The behavior of rats at baseline and before euthanasia was assessed by Zausinger' 6-point scale neural deficit scores [17] as previously reported which was performed by a single researcher who was blind to the experimental groups. The scale was carried out as follows: (0), without spontaneous activity; (1), falling to the contralateral side; (2), severe circling when tail pull; (3), lowered resistant to contralateral push; (4), unable to extend the contralateral forelimb; (5), no deficit. The lower the score is, the worse the neural functional lesion is.

### 2.6. Infarction Volume Assessment

2,3,5-Triphenyl tetrazolium chloride (TTC) staining was adopted to quantify the infarction volume as previously reported [13]. Briefly, brains were quickly removed after decapitation and then frozen in −20°C for 30 min. The frozen brains were sectioned equally into 5 pieces (about 2 mm each). All sections were stained with 0.4% TTC (Sigma, USA) solution for 20 min at 37°C followed by fixation in 12% formalin solution for 2 min. Then the TTC stained slices were photoed by Olympus fe-240 digital camera (Pooher Photoelectric Technology Co., Ltd., Shanghai, China) and analyzed by Image Analysis Software (Image-pro Plus 6.0, Media Cybernetics, Inc., Bethesda, MD, USA). The infarction volume was presented as a percentage of the total ipsilateral hemispheric volume which can be calculated by following equation: [(contralateral hemispheric volume − ipsilateral hemispheric volume)/contralateral hemispheric volume] × 100% [18, 19].

### 2.7. Cerebral Blood Flow Observation

A laser-Doppler flow meter was employed for observing cerebral blood flow in cerebral pia mater by using a flexible fibre optic to monitor moving red blood cells. In brief, rat's head was secured in a stereotactic frame under anesthesia by 10% chloral hydrate (250 mg/kg) intraperitoneal injection. A center incision was made to expose the skull. A 1.0 mm × 2.00 mm hole ahead of the bregma was carefully drilled by a dental drill while superfused by warm saline. Then the cerebral blood flow was measured by a laser-Doppler flow meter (Moor-DRT4, Wilmington, DE, USA). The laser-Doppler probe recorded the cerebral blood flow on intact dura mater for 1 minute. Data was analyzed by bundled software.

### 2.8. Immunofluorescence Double-Staining

Immunofluorescence double-staining was employed to inspect the endogenous neurogenesis with antibodies against BrdU and nestin. Paraffin-embedded sections were dewaxed in dimethylbenzene and hydrated in gradient ethanol after heating at 70°C for 2 hours, followed by antigen retrieval in citrate buffer (pH = 6.0) in microvan at medium heating for 10 min and cooled in room temperature for 1 hour. Slices were then washed in 0.01 M PBS 3 times for 5 minutes each time, subsequently despiralized in 2 N HCl (1 : 5) for 30 minutes at 37°C and blocked in 10% goat serum for 30 minutes. The blocked slices were then incubated in BrdU and nestin combination primary antibodies which dissolved in 0.01 M PBS with the dilution data 1 : 50 and 1 : 100, respectively, overnight at 4°C. And the secondary antibody Goat anti-Mouse AlexaFluor 488 (l : 300) and Goat anti-rabbit AlexaFluor 594 (l : 300) were used on the second day. Negative controls received the same treatment omitting the primary antibodies and showed no specific staining. 5 visual regions in penumbra were selected for immune positive cells counting under a 400x microscope.

### 2.9. Real-Time Polymerase Chain Reaction

The cortex, hippocampus, and striatum of rats brain were rapidly dissected and total RNA was purified by using Trizol Reagent (Invitrogen, USA) according to the manufacturer's instructions. The complementary DNA was synthesized by Shanghai Science & Technical Co. (Shanghai, China). Forward and reverse primers were 5′-CTCTTGGCTTTCTGGACCCC-3′ and 5′-CACAGGAGTCTCAAGGGTATTAGGC-3′ for nestin, 5′-CAGCCTTCCTTCCTGGGTATG-3′ and 5′-TAGAGCCACCAATCCACACAG-3′ for actin. All samples were normalized by actin. Each sample was tested in triplicate. Relative gene expression was measured as 2^−ΔΔCT^ method [20].

### 2.10. Microarray Analysis

To observe the effects of acupuncture treatment on gene transcription after ischemia, microarray analysis was employed. Ipsilateral brains were collected and stored in liquid nitrogen preparing for microarray analysis. Total RNA was extracted using a Trizol reagent (Invitrogen, USA) according to the manufacturer's instructions and quantified by ultraviolet spectrophotometer and polyacrylamide gel electrophoresis. Purified RNA was converted to cDNA and amplified by using Illumina TotalPrePRNA kit (Illumina, USA). Followed sample labeling, hybridization (Gene Expression Hybridization Kit Agilent p/n 5188–5242), feature extraction (Agilent G4450AA Feature Extraction software 10.7), and image scanning (Agilent Scan Control software) were utilized Agilent standard protocol. Briefly, degenerated hybridization solution was sampled on slides followed by hybridized in hybridization chamber for 1~2 hours at 42°C as prehybridization. Mixture combined reverse transcription product and hybridization solution was degenerated in 95°C for 2 minutes while prehybridized slides degenerated in 95°C for 30 seconds. Then, target DNA in chips was hybridized at 42°C overnight. After hybridization, the arrays were washed twice for 10 minutes each in washing solution. Fluorescence signals from each microarray were collected by DNA microarray scanner and converted to original data. The data was preprocessed by subtract in limma package software. Probe expression in chips was represented as mean. Gene expression values between groups that increased by 2-fold or decreased by 0.5-fold were considered to be significant difference (*P* < 0.005). Gene Ontology (GO) analysis was employed to analyze functional enrichment. Pathway enrichment analysis was utilized to analyze involved pathway by searching in KEGG (Kyoto encyclopedia of Genes and Genomes) data base. Expression profile chip in each group had 5 times biological repeat.

### 2.11. Enzyme-Linked Immunoabsorbent Assay (ELISA)

The expression of GSK-3*β* and PP2A was evaluated by enzyme-linked immunoabsorbent assay. Ipsilateral brains from each group were divided into cortex, hippocampus, and striatum after decapitation. The tissue was homogenized and purified by centrifugation. GSK-3*β* and PP2A Immunoassay ELISA kit (R&D, USA) were used to determine GSK-3*β* and PP2A level in supernatants according to the manufacturer's instructions. Absorbance of each sample was measured using a microplate reader at a wavelength of 450 nm. All the samples were measured in duplicate.

### 2.12. Statistical Analysis

All data were analyzed by SPSS 17.0 software and present as Mean ± SD except for NDS and microarray. One-way ANOVA followed by LSD and a post-hoc Mann-Whitney *U* test was used for analyzing the data. The standard statistical function of R/bioconductor, *t*-test, F-criterion of ANOVA, and FDR was performed for determined genes differential expression of microarray analysis. Possibility values of <0.05 were considered as statistically significant.

## 3. Results

### 3.1. Acupuncture Alleviated Neural Functional Deficit

**Figure 2:**
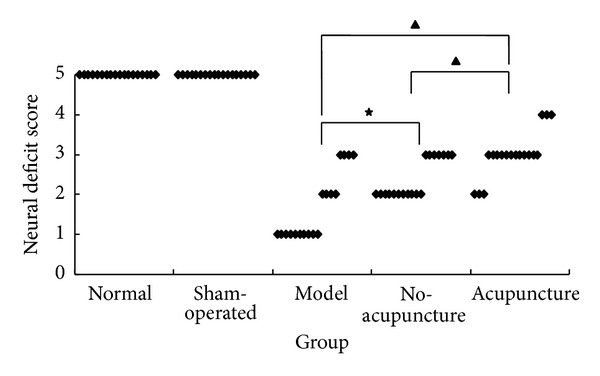
Acupuncture attenuated neural functional deficit. Neural deficit score (NDS) was employed for evaluating the neural functional deficit (*n* = 18). The lower the score is, the worse the damage is. There were significant differences among MCAO groups: no-acupuncture group got higher scores than model group (★*P* < 0.05) suggesting a “self-repairing” ability of MCAO rats. Acupuncture group got the highest scores in MCAO group while lower scores in model and no-acupuncture group (▲*P* < 0.05) which meant that acupuncture attenuated neural damage after MCAO but did not reach to a normal level.

Neural functional deficit was evaluated by Zausinger et al. [17] 6 point scale at baseline and before euthanatized in MCAO groups and the corresponding time point of normal and sham-operated group. Mann-Whitney *U* test was conducted to analyze the neural deficit score. As shown in Figure 2, there were significant differences among MCAO groups and normal, sham-operate group (*P* < 0.05) which means MCAO caused a dramatic damage on neural function. Compared two no-intervention groups, no-acupuncture group shown an attenuation of neural damage to model group suggesting a self-repairing capacity of rat post ischemia infarction. But acupuncture group shown a distinct increased in NDS compared to other MCAO groups while still lower than normal and sham-operated group indicating that acupuncture relieves the neural functional damage but did not reach to a normal level.

### 3.2. Acupuncture Improved Cerebral Blood Flow

**Figure 3:**
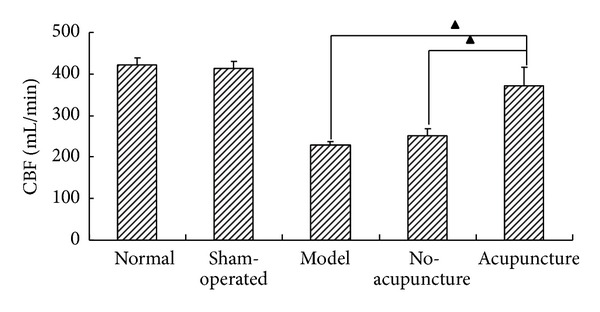
Acupuncture improved cerebral blood flow (CBF). A sharp decrease in cerebral blood flow was found in model group and no-acupuncture group compared to normal and sham-operated group. No significant difference was found between model group and no-acupuncture group. But acupuncture increased the cerebral blood flow induced by MCAO (▲*P* < 0.05) (*n* = 6).

As shown in Figure 3, cerebral blood flow in rats of model group and no-acupuncture group were sharply decreased after onset of MCAO. In contrast, there was a significant increase of CBF in acupuncture group compared to model group and no-acupuncture group, suggesting the shortage of brain blood supply caused by middle cerebral artery occlusion could be rectified by acupuncture which approximately reached to the normal level.

### 3.3. Acupuncture Did Not Decrease the Infarction Volume

**Figure 4:**
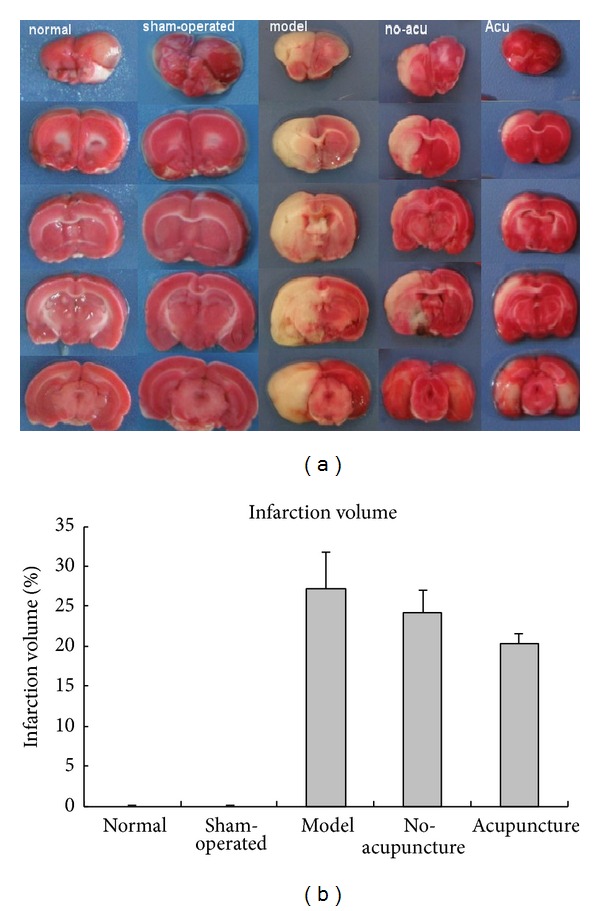
Observation of infarction volume: (a) representative coronal sections stained by TTC from five groups (*n* = 6), (b) quantitative analysis of infarction volume. MCAO caused obvious infarction in MCAO groups compared to normal and sham-operated group. Although acupuncture group shown a tendency of decrease in infarction volume compared to model group and no-acupuncture group, no significant difference has been found.

To observe neural protective effect of acupuncture on MCAO rats, TTC staining was utilized for evaluating the infarction volume. Conspicuous infarction formed in brain of rats in model group, no-acupuncture group, and acupuncture group (shown in Figure 4(a)), whereas no infarction observed in normal and sham-operated group. No marked difference was found between model group and no-acupuncture group. There was a tendency of decrease in acupuncture group compared to model group and no-acupuncture group but no significant difference has been found.

### 3.4. Acupuncture Enhanced the Neurogenesis in MCAO

Immunofluorescence double-staining combined with real-time polymerase chain were adopted for examining the endogenous neurogenesis. BrdU co-labeled with nestin (a special marker for neural stem cells in central nervous system (CNS)) were employed for labeling neural stem cells [21]. Furthermore, RT-PCR was used to quantify the mRNA expression of nestin in cortex, hippocampus, and striatum, respectively.

**Figure 5:**
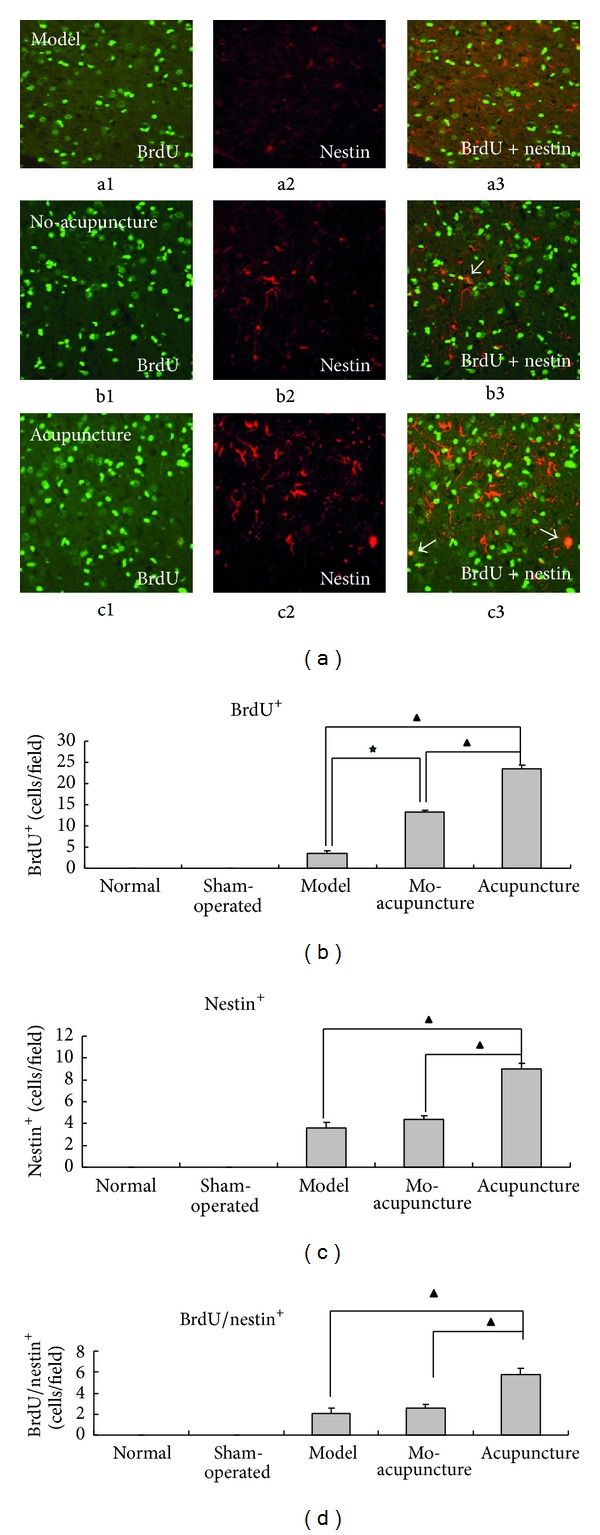
Effects of acupuncture on increasing population of immunopositive cells in penumbra: 5 visual regions in penumbra of each slice were selected for cell counting under a 400x microscope (*n* = 6). (a) Representative images of Immunofluorescence double-staining from model group, no-acupuncture group, and acupuncture group: a1, b1, and c1 labeled for BrdU (green). a2, b2, and c2 labeled for nestin (red). a3, b3, and c3 colabeled for BrdU/nestin. White arrows points to the BrdU/nestin^+^ cells. (b), (c), and (d) Quantization of BrdU^+^, nestin^+^, and BrdU/nestin^+^ cells: immunopositive cells were found in MCAO groups while not been found in normal and sham-operated group. Acupuncture enhanced BrdU^+^, nestin^+^, and BrdU/nestin^+^ labeled cells proliferation (▲*P* < 0.05, acupuncture group versus model group and no-acupuncture group). More BrdU^+^ labeled cells in no-acupuncture group than model group (★*P* < 0.05).

By immunofluorescence double-staining, a large number of BrdU^+^, nestin^+^, and BrdU/nestin^+^ cells in MCAO groups were observed as shown in Figure 5, whereas no immune-positive cells were found in normal and sham-operated group. There was also a significant increase of immunopositive cells in acupuncture group versus model group and no-acupuncture group (shown in Figures 5(b), 5(c), and 5(d)), suggesting acupuncture treatment induced proliferation of neural stem cells in a certain extent. And BrdU^+^ cells obviously increased in no-acupuncture group compared to model group. However, no distinct difference was found in nestin^+^ and BrdU/nestin^+^ between no-acupuncture group and model group (shown in Figures 5(c) and 5(d)).

**Figure 6:**
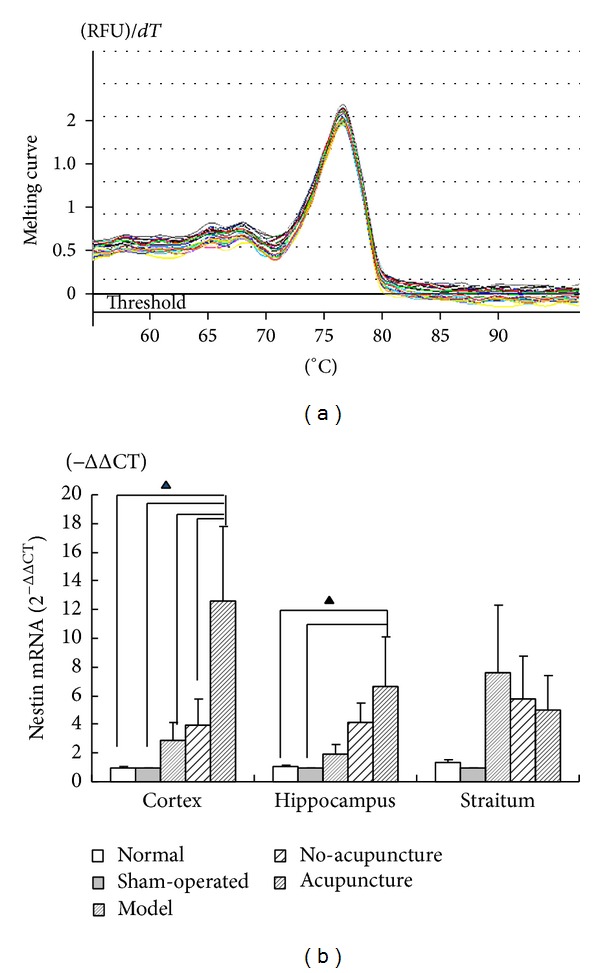
Analysis of the mRNA expression of nestin by real-time PCR: (a) melting curve of nestin by real-time PCR, (b) relative expression of nestin mRNA in cortex, hippocampus, and striatum (2^−ΔΔCT^) shows in bar graphs: rare expression of nestin was found in normal and sham-operated group in brain. Nestin mRNA expression of model group greatly increased in striatum and slightly increased in cortex followed by hippocampus. However, expression tendency was different in no-acupuncture group: enhanced in cortex and hippocampus but suppressed in striatum which supposed to be a “self-repairing” control group. Acupuncture group got the highest expression of nestin mRNA in cortex versus other MCAO groups (▲*P* < 0.05) while higher in hippocampus (▲*P* < 0.05) and less in striatum.

Consistent with the results of immunofluorescence double-staining, model group shown a high expression of nestin mRNA compared to normal and sham-operated group especially in striatum followed by hippocampus and cortex suggesting there was different expression tendency in three cerebral regions after ischemia. However, nestin expression of no-acupuncture group increased in cortex and hippocampus but decreased in striatum (shown in Figure 6). As to model group, no-acupuncture group supposed to be a “self-repair” control group, because neither of them received any intervention but sacrificed at 3 days after the model group. From nestin expression of cortex, hippocampus, and striatum, we found that different cerebral regions shown different “self-repair” capacity. And the “self-repair” capacity could be enhanced by acupuncture stimulation; as demonstrated in Figure 6, nestin mRNA was significantly increased in cortex while slightly increased in hippocampus and decreased in striatum compared to model group and no-acupuncture group. Cortex may be more sensitive for acupuncture stimulation than hippocampus and striatum.

### 3.5. Acupuncture Treatment Evoked Expression of Phosphorylation and Cell Proliferation Relative Genes

To obtain the gene expression stimulated by acupuncture treatment after ischemia, we performed a microarray analysis to identify differentially expressed genes. According to analytic strategy mentioned above (*P* adjust ≤0.01), we first determined 12350 differentially expressed genes among normal, sham-operated, model, no-acupuncture, and acupuncture groups. All significantly and differentially expressed genes were analyzed by Gene Ontology (GO) analysis to identified relative biological process, cellular component, and molecular functions. As shown in Table 1, total of 444 biological processes, 164 molecular functions, and 47 cellular components were involved in the physiopathological processes. And the top 10 enriched GO biological processes were included such as tyrosine phosphorylation, immunologic tolerance, protein negative regulated, and cell proliferation. Additional, KEGG analysis was employed for pathway enrichment. The top 10 enriched pathways were involved in neural signaling transduction, glutamate metabolism, neuroactive ligand-receptor interaction, complement and coagulation cascades, biosynthesis of steroids, and cytokine-cytokine receptor interaction as demonstrated in Table 2. In general, the differentially expressed genes regulated by acupuncture were mainly belonging to biological processes of metabolism, phosphorylation, cell proliferation, and neural signaling transduction.

### 3.6. Acupuncture Made a Positive Regulation on GSK-3*β*, PP2A, and G/P

**Figure 7:**
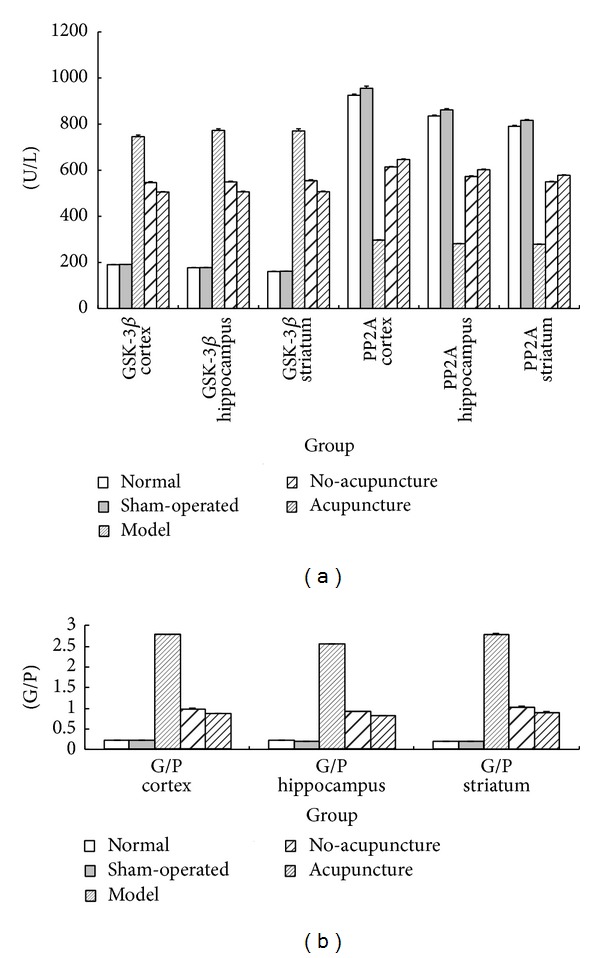
Acupuncture made a positive regulation of GSK-3*β*, PP2A, and G/P. GSK-3*β* expression and G/P significantly increased in MCAO groups but decreased in acupuncture group (*P* < 0.05). PP2A expression showed a reverse tendency. The expression of GSK-3*β* and PP2A had a temporal profile. But acupuncture had a positive regulation for GSK-3*β*, PP2A expression, and G/P (*P* < 0.05).

Microarray analysis was shown biological processes of metabolism, phosphorylation, and cell proliferation relevant to neural protective effect after ischemia. Thus, to verify the microarray analysis results, we evaluated GSK-3*β* and PP2A expression (which well-accepted have closely connection of biological processes mentioned above) in different cerebral regions of rats by ELISA. As demonstrated in Figure 7(a), GSK-3*β* expression robust increased in MCAO groups, whereas PP2A expression conspicuous decreased. And expression of GSK-3*β* in no-acupuncture was decreased meanwhile increased of PP2A expression compared to model group (*P* < 0.05). However, acupuncture shown a regulation effects on expression tendency of GSK-3*β* and PP2A: it enhanced the increasing of GSK-3*β* while attenuated decreasing of PP2A (*P* < 0.05). And the similar tendency of GSK-3*β* and PP2A expression was found in cortex, hippocampus and striatum. The ratio of GSK-3*β* and PP2A was also dramatically elevated post-ischemia, but decreased in no-acupuncture group and acupuncture group (*P* < 0.05). The results revealed that it was a temporal profile of GSK-3*β* and PP2A expression, and the ratio of GSK-3*β* and PP2A (G/P) positive regulated by acupuncture stimulation.

## 4. Discussion

In the present investigation, we gave experimental evidences that acupuncture treatment enhanced the “self-repairing” capacity of MCAO rats to alleviate neural functional damage induced by ischemia via multiple outcome measurements. Using laser-Doppler flow meter, double-label immunostaining, RT-PCR, microarray and ELISA, we observed that acupuncture obviously increased brain blood flow (Figure 3), neurogenesis (Figure 5) and expression of nestin mRNA (Figure 6), and caused a series changing of genes and GSK-3*β*/PP2A (proteins that are relative to biological processes of phosphorylation and cell proliferation) expression (Table 1 and Figure 7).

Although pathological and pathophysiological in brain after ischemia stroke is quite complicated [22], it's ascertaining that endogenous neurogenesis and angiogenesis contribute to neural functional rehabilitation in special area of CNS [23]. As evidences given by recently extensive reports, the beneficial effects of acupuncture on brain ischemic damage in vivo or in vitro mainly focus on microcirculation [24], anti-apoptotic [25], anti-inflammation [26], neuron protection [27], brain metabolism [28]. Our previous studies indicated that acupuncture combined with basic modern medicine treatment showed a great effect on stroke patients' self-care ability and quality of life in clinic trial [12], and the underlying mechanism may be associated with improving cerebral haemodynamics and neuron protective effect under a special stimulated parameter on a special acupionts revealed by animal researches [13, 29, 30].

In present study, we utilized the optimum parameter (3 times per second for 1 minute) as proved by previous study for acupuncture stimulation on DU26 [31]. We found out that cerebral blood flow dramatically increased in acupuncture group. As we know, cerebral blood supply in correspond area is significantly decreased after middle cerebral artery occluded, followed by vulnerable neurons apoptosis rapidly in such a hypoxic-ischemic environment. However, acupuncture increasing the cerebral blood supply in ischemia region is helpful to ameliorate the hypoxic-ischemic situation to a certain extent which may be relevant to regulating vascular caliber, establishing collateral circulation and enhancing angiogenesis [29, 32].

Except for regulating focal cerebral hemodynamics, neurogenesis also has been observed in acupuncture treatment group. BrdU/nestin co-labeled immunofluorescence positive cells and mRNA expression of nestin were detected for evaluating neurogenesis in this study. BrdU is usually used to investigate cell proliferation as it integrates with cell circle during DNA synthesis phase which is considered as a “Gold standard” to determine neurogenesis [16]. In present research, we have chosen the seventh day after MCAO surgery to observed neurogenesis for a better integration of BrdU into DNA. Nestin is one of intermediate filament proteins that is abundantly and transiently expressed in neural stem cells of developing and developed central neural system and has always been utilized for identifying undifferentiated CNS precursor's proliferation [33–35]. Many BrdU^+^, nestin^+^, BrdU/nestin co-labeled immunofluorescence positive cells were seen in model group in penumbra belt in the study. These endogenous neurogenesis amplified by acupuncture stimulation showed a significant increase of immuno-positive cells in acupuncture group compared with model and no-acupuncture group (Figures 5(b), 5(c), and 5(d)). But it is well accepted that generation of neurogenesis only occurs in special regions such as SVZ and SGZ in rodents and humans as response to neuron injury [36, 37], therefore immuno-positive cells in penumbra may migrate from the specific neurogenesis regions and differentiate to neurons or glial cells for subsequent functional recovery. As to nestin, acupuncture showed a strong effect on its mRNA expression. Nestin expression in brain was obviously elevated by ischemia infarction, but different tendency were found in cortex, hippocampus and striatum. High expression of nestin mRNA in cortex was coincident with some previous reports which presented in some neurons or neurogliocyte localized in boundary zone of infarction area and showed a temporal profile [34, 38, 39]. We consider these temporal neurogenesis phenomenons accompanied by alleviation of neural functional damage in model group and no-acupuncture group as “self-repairing”. It means focal ischemia damage could be in spontaneous remission to some extent even without any intervention. Among three brain regions, striatum got the highest expression immediately after MCAO, followed by cortex and hippocampus. However, “self-repairing” capacity in MCAO rats' brain reversed such tendency as mentioned above 3 days after MCAO: Nestin expression in cortex and hippocampus continued to increase while it decreased in striatum. Robustly increasing nestin expression in cortex demonstrated that acupuncture could enhance the “self-repairing” capacity, (while nestin expression slightly increased in hippocampus and decreased in striatum). Thus, we speculate that acupuncture stimulation on DU26, who is the key acupoint of* Xing Nao Kai Qiao *acupuncture therapy prescription, may influence on facilitating neural stem cells migration and enhancing “self-repairing” capacity in MCAO rats.

Intriguingly, compared with model group, improvement of neural functional damage found in no-acupuncture group and acupuncture group were not accompanied by reduction in infarction volume, which suggests that neural functional improvement did not correspond to significant decrease of infarction volume. That may be because only little part of newborn stem cells survived which is too few to reach morphological changing [8], but survived newborn stem cells had participated in neural circuit to function.

Given the results mentioned above, we speculate that acupuncture may achieve neuroprotection by enhancing self-repairing capacity which increases cerebral blood flow to create an appropriate microenvironment for subsequent proliferation and migration of neural stem cell towards the ischemic boundary region to replenish damaged neuron. However, it is a long process for a neural stem cell to develop into a fully functional neuron. It includes neural stem cell generation, progenitor cell amplifying, differentiation, migration, synapses and neural circuit formation in a preexisting neural system. To determine the exact role of acupuncture in alleviation of neural damage in MCAO, microarray analysis was employed. Microarray analysis is helpful to identify groups of genes distinctively changed among groups. The microarray results revealed that many genes and pathways were involved which were mainly relevant to phosphorylation, immunologic tolerance, protein regulation and cell proliferation functions by GO and pathway analysis. From the result of microarray assay, we found out that acupuncture treatment partly took effect by enhancing “self-repairing” capacity but was also mediated by gene transcription regulation involving many biological processes. All of them have potential for exploring ischemia treatment strategy.

To verify the results of microarray, measurement of protein GSK-3*β* and PP2A which play a crucial role in the above biological process was conducted. Glycogen synthase kinase-3*β* (GSK-3*β*) and protein phosphatase2A (PP2A) are important regulators of protein phosphorylation in many pathway. Protein phosphorylation known to a key regulation mechanism of cellular signal transduction is activator for neurons function as protein regulation and proliferation by *β*-catenin or MARK pathway. GSK-3*β*, initially considered as a regulator of glycogen metabolism highly expressed in CNS [40], now is important in controlling protein synthesis, cell proliferation, differentiation and apoptosis [41]. PP2A negatively regulate GSK-3*β* and signal transduction of subsequent pathway by dephosphorylation. Mediation balance of GSK-3*β* (upregulate) and PP2A (downregulate) expression have been associated with regulation of CNS neurons. Here, we found out that high expression of GSK-3*β* and suppression of PP2A were evoked by ischemia damage but regulated by acupuncture treatment in vivo, which suggests that mechanism of acupuncture neuroprotection may be associated with regulation GSK-3*β* and PP2A expression. However, further study is needed to determine which part is exactly participating in regulation of endogenous neurogenesis and genes expression changing post-ischemia damage.

Taken all the results together, we found out that the mechanism of acupuncture therapeutic effect on focal ischemia infarction is extremely complex. It is not only regulated by one single or two elements but involved multiple targets to achieve neural protective effect. The concept is consistent with old system theory of Traditional Chinese Medicine which is distinct from reductionism of modern medicine. The integrative system theory holds that human has a balance system that consists of many organs, and also a part of society and environment. Thus, multiple strategies are needed to diseases treatment. The* Xing nao kai qiao* acupuncture prescription consists of DU26, P6, SP6, LI11 and B40, established by Dr. Xuemin Shi for stroke treatment in 1970's. He holds that the main pathogenesis of stroke is “blockage of brain lead to spirit away”. The spirit is the impetus of life activities in traditional medicine theory who is* qi *in essence governed by brain. It governs human life activities through* qi* from brain combine* qi* from kidney together then to trigger other organs'* qi*. Thus, the treatment principle of stroke should unblock the blockage in brain and promote circulation of* qi* in body. Here, we selected DU26 (locating at the upper one-third and lower one-third of the cleft lip midline) in the prescription for it belong to the governor meridian that is traveling through brain and govern* qi* of body. It also follows the nearby acupuncture prescription principle for stroke locating on brain. And, modern anatomical study show that its skin innervates by trigeminal and deeper muscle innervates by facial nerve. So stimulating DU26 means stimulate trigeminal and facial nerve directly and the nerve impulse conduct to nucleus nervi facials and trigeminal nerve nuclei in brainstem. Confined by methodology, we have to study one acupoint each time, so we stimulated DU26 to determine the underlying mechanism of acupuncture on ischemia damage. Thus, more researches are needed.

In conclusion, the present study indicated that the underlying mechanism of acupuncture treatment on neural functional damage caused by focal ischemia injury was a multiple action which may be associated with improved cerebral blood supply, neurogenesis and regulation of transcription or GSK-3*β* and PP2A expression in MCAO rats. Acupuncture is a potential therapeutic strategy for ischemia stroke.

## Figures and Tables

**Table 1:** The top 10 enriched GO BP of the differentially expressed genes.

GO ID	GO term	*P*-value	Gene name
GO:0042506	Tyrosine phosphorylation of Stat5 protein	0	Jak2;Ptk6;Osm;Jak3
GO:0042503	Tyrosine phosphorylation of Stat3 protein	0	Jak2;Ptk6;Lif;Osm
GO:0002513	Tolerance induction to self-antigen	0	Lyn;Foxp3;Tgfb1
GO:0045759	Negative regulation of action potential	0	Chrnb2;Cnr2;Cnr1
GO:2000009	Negative regulation of protein localization at cell surface	0	Tax1bp3;Gpm6b;Leprot
GO:0090191	Negative regulation of branching involved in ureteric bud morphogenesis	0	Six1;Tacstd2;Grem1;Bmp4
GO:0090197	Positive regulation of chemokine secretion	0	Il33;C5;Csf1r;Il4ra;Chia;Il1rl1
GO:0045356	Positive regulation of interferon-alpha biosynthetic process	0	Tlr7;Tlr9;Tlr3
GO:0072110	Glomerular mesangial cell proliferation	0	Pdgfb;Pdgfrb;Egr1
GO:0048148	Behavioral response to cocaine	0	Drd2;Drd1a;Abat;Crhr1;Adra1b;Snca;Drd3;Drd4;Cdk5

**Table 2:** The top 10 enriched pathways of the differentially expressed genes.

KEGG ID	KEGG pathways	*P* value	Gene name
4740	Olfactory transduction	0	Olr841;Olr847;Olr845;Olr853;Olr852;Olr851;Olr850;Olr848;Olr 1006;Olr1007;Olr1660;Olr878;Olr1057;Olr1012;Olr1658;Olr936; Olr950;Olr943;Olr947;Olr951;Olr1002…

471	D-Glutamine and D-glutamate metabolism	0	Gls;Glud1;Gls2

4080	Neuroactive ligand-receptor interaction	3.46*E* − 12	Lpar2;Prss1;Prl;Prlr;Ptgfr;Tshb;Adrb3;Glra1;Cckbr;Mc5r;Chrng; Prss3;Hrh1;Htr1a;Lepr;Glra2;Gh1;Ednra;Drd2;Drd1a;Lpar3;Npy 2r;LOC286960;Grik4;Grin1;Grm3;Grm1;Grin2c…

4610	Complement and coagulation cascades	4.52*E* − 06	Plat;Serpina1;Serpine1;C1qc;Mbl1;Serpinc1;F5;Fgg;Fgb;Pros1;F ga;Thbd;Kng1;F9;Bdkrb1;Plaur;C1s;Vwf;C3ar1;C7;C9;C5;Cr1l; Cfd;Cfh;C8g;Serpina5;C1r;Serping1;F7;Cd55;Masp1;F13a1;Cr2; Serpind1;Kng2;Klkb1;Masp2;F8;A2m;F13b;C4bpa;C4a;C6;C2;C 3;Cpb2;C5ar1;F10;Mbl2;C1qa;F2r;Plau;F3;Proc;Bdkrb2;C1qb;C 8a

5150	Staphylococcus aureus infection	4.68*E* − 06	Selp;C1qc;Mbl1;Fcgr3a;Fgg;RT1-Ha;Fcgr2a;C1s;Dsg1b;Krt10;C 3ar1;C5;Fcar;Fcgr1a;Cfd;Ptafr;Cfh;C1r;Masp1;LOC688090;Itga m;Masp2;Fcgr2b;Fpr2l;Itgb2;RT1-Ba;RT1-Bb;RT1-DMa;RT1-D Mb;RT1-Db1;RT1-Da;C4a;C2;C3;C5ar1;Selplg;Mbl2;C1qa;Icam 1;LOC498276;Il10;C1qb;Itgal

4640	Hematopoietic cell lineage	6.65*E* − 06	Il7;Mme;Il2ra;Cd3d;Il6;Il5;Il6r;Il1a;Il1b;Il3;Il9r;Epo;Epor;Itga6; Anpep;Cd36;Cd37;Il3ra;Cd9;Cd8a;Cd8b;Cd4;Ms4a1;Tnf;Il7r;Csf 2;Gp1bb;Cd19;Csf1;RGD1565355;Il5ra;Fcgr1a;Gp9;Flt3;Itga5;K itlg;Itga2;Tpo;Il1r2;Cd3e;Csf1r;Kit;Cd55;Csf2ra;Cd14;Cr2;Cd3g; Il4ra;Itgam;Cd24;Cd7;Cd1d1;Itgb3;LOC687856;RT1-Db1;RT1-D a;Thpo;Csf3r;Cd44;Dntt;Csf3;Il4;Il11;Fcer2;Gp5

100	Biosynthesis of steroids	6.69*E* − 05	Cyp27b1;LOC691221;Soat1;Tm7sf2;Cyp2r1;Nsdhl;Sc5dl;Ebp;D hcr7;Hsd17b7;Lipa;Fdft1;Cel;Sqle;Cyp51;Msmo1;Soat2;Dhcr24

4742	Taste transduction	7.05*E* − 05	Scnn1g;Scnn1b;Itpr3;Kcnb1;Gnat3;Gnb1;Grm4;Gng13;Gnas;Tas 2r137;Tas2r138;Tas2r126;Trpm5;Tas2r108;Tas2r102;Tas2r114;Ta s2r121;Tas2r118;Tas2r107;Tas2r13;Tas2r105;Tas2r119;Tas2r144; Gng3;Tas2r140;Adcy4;Tas2r143;Tas2r135;Prkx;Plcb2;Tas1r3;Tas 2r134;Tas2r123;Tas1r1;Scnn1a;Tas2r120;Adcy8;Cacna1b;Tas2r1 39;Adcy6;Tas2r136;Asic2

5323	Rheumatoid arthritis	7.35*E* − 05	Cxcl12;Tnfsf13b;Cd28;Ctsl1;Ifng;Acp5;Il6;Ccl12;Il1a;Il1b;Jun;L tb;Vegfa;Ctsk;Ccl5;Il18;Tcirg1;Atp6v1a;Atp6ap1;RT1-Ha;Tnf;At p6v0d1;Atp6v1e2;Tlr2;Atp6v1g2;Atp6v1f;Csf2;Tnfsf11;Atp6v1b 2;Csf1;Cd86;Flt1;Il23a;Mmp1a;Cxcl5;Atp6v0c;Atp6v1b1;Atp6v 1h;LOC688090;Atp6v1g3;Fos;Atp6v0a4;Ctla4;Ccl20;Tgfb1;Atp 6v0e1;Itgb2;RT1-Ba;RT1-Bb;RT1-DMa;RT1-DMb;RT1-Db1;RT 1-Da;Atp6v0e2;Tlr4;Cd80;Icam1;Tnfsf13;Angpt1;Ccl3;Il11;Mm p3;Tnfrsf11a;Itgal;Il17a

4060	Cytokine-cytokine receptor interaction	0.000178	Prl;Prlr;Cxcl12;Il22;Tnfsf13b;Il7;Met;Pdgfrb;Cntf;Il2ra;Ifng;Il2r b;Il28ra;Il22ra1;Il6;Il5;Ccl12;Il6r;Il1a;Il1b;Il3;Ifnb1;Il9r;Ccl28; …
